# Singing and Calling Behavior of a Threatened Passerine With Spatially Variable Vocalizations: Individual, Population and Geographic Patterns

**DOI:** 10.1002/ece3.74380

**Published:** 2026-09-18

**Authors:** Cristian Pérez‐Granados, Cristina D. Alonso‐Moya, Adrián Barrero, Pedro Sáez‐Gómez, Gerard Bota, José J. Lahoz‐Monfort, Paola Laiolo, María Méndez, David Serrano, José L. Tella, Matthias Vögeli, Juan Traba

**Affiliations:** ^1^ Biodiversity Management and Conservation Programme, Forest Science and Technology Centre of Catalonia, CTFC Lleida Spain; ^2^ Terrestrial Ecology Group (TEG‐UAM), Departamento de Ecología Universidad Autónoma de Madrid Madrid Spain; ^3^ Centro de Investigación en Biodiversidad y Cambio Global (CIBC‐UAM) Universidad Autónoma de Madrid Madrid Spain; ^4^ Department of Ecology and Evolution Estación Biológica de Doñana (CSIC) Seville Spain; ^5^ Instituto Pirenaico de Ecología, CSIC Jaca Spain; ^6^ School of Agriculture, Food and Ecosystem Sciences University of Melbourne Parkville Victoria Australia; ^7^ Instituto Mixto de Investigación en Biodiversidad (CSIC, UO, PA), Universidad de Oviedo, Campus de Mieres Mieres Spain; ^8^ German Center for Integrative Biodiversity Research (iDiv) Halle‐Jena‐Leipzig Leipzig Germany; ^9^ Institute of Biology Martin Luther University Halle‐Wittenberg Halle (Saale) Germany; ^10^ Departamento de Biología de la Conservación y Cambio Global Estación Biológica de Doñana (EBD‐CSIC) Sevilla Spain; ^11^ Swiss Ornithological Institute Applied Ecology Research Unit Sempach Switzerland

**Keywords:** acoustic signal, birds, *Chersophilus duponti*, Monte Carlo, repertoire size, song sharing, vocal behavior

## Abstract

Acoustic communication plays a central role in bird behavior, yet the ecological and social factors that shape the patterns of variable within‐species bird vocalizations remain poorly understood. Here, we examined the singing and calling behavior of Dupont's lark ( *Chersophilus duponti* ), a threatened bird species, across individuals, populations, and spatial scales throughout most of its European distribution range, restricted to Spain. For both vocalization types, we assessed the influence of population size and recording location on individual and population repertoire sizes, described the spatial patterns of acoustic similarity among males, and tested whether individual song repertoire predicts population persistence, a hypothesis formulated for this species two decades ago. We found that individual song repertoire size differed between the two geographic areas surveyed, with males inhabiting the Ebro Valley having a larger song repertoire size than those in the Iberian System, but this was unrelated to population size. Call repertoire size showed no relationship with either factor. At the population level, song repertoire size increased with population size, whereas call repertoire size was not associated with any considered driver. Acoustic similarity in both vocalization types decreased with spatial distance, but songs exhibited a sharper spatial decay than calls, suggesting more restricted cultural transmission. Mean individual song repertoire size was a significant predictor of population persistence over two decades: populations in which individuals had larger song repertoires in 2004–2007 were significantly more likely to persist to the present day, supporting a long‐standing hypothesis for this species. Overall, our results reveal distinct ecological and cultural signatures for songs and calls in Dupont's lark. This indicates that songs, which are learned after dispersal, are more dynamic and variable, whereas calls, which are proposed to be learned before dispersal, are more stable. Furthermore, we confirm that song repertoire size can be used as an early‐warning signal of population decline and highlight the utility of acoustic traits as practical tools for tracking population health and informing conservation strategies.

## Graphical abstract

Singing and calling behavior of birds may differ between individuals, populations and landscape features.

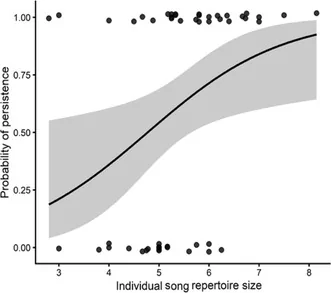

## 1 Introduction

Acoustic communication is widespread across wildlife and plays a fundamental role in mediating interactions both within and across species. Bird vocalizations constitute one of the most diverse communication systems, encoding information related to species and individual identity, individual quality, and behavioral context, among others (e.g., Nowicki et al. 1998; Farrow et al. 2017; Knight et al. 2024). In fact, acoustic signals of birds are central to key behaviors such as mate attraction, territorial defense, defense from predators, and social cohesion (Todt and Naguib 2000; Catchpole and Slater 2008), thereby directly influencing fitness (Hiebert et al. 1989, but see Slabbekoorn and Smith 2002). In recent years, the study of bird vocalizations has also gained momentum as both conservation and monitoring tool, revealing how habitat fragmentation and environmental changes shape communication networks and population connectivity (Laiolo 2010; Lewis et al. 2021), as well as demonstrating the potential of acoustic data for effective biodiversity monitoring (Bell and Malerba 2025). Despite over a century of research on avian acoustics (i.e., Wright 1912; Allen 1913), there is still much to be understood about the ecological, evolutionary, and behavioral forces that shape spatial structure of bird vocalizations, as well as how this knowledge can inform conservation strategies.

One reason for this lack of knowledge for most avian vocalizations relies on the wide diversity of vocalization types, each associated with distinct functions, learning processes and contexts. Bird vocalizations are often divided into songs and calls, which can also be classified as territorial, warning, group, distress, and begging calls (Catchpole and Slater 2008), among others. Songs are typically more complex and linked to sexual selection and learning processes, whereas calls are often shorter, more innate, and associated with territorial defense or immediate communicative purposes (Catchpole and Slater 2008). These differences suggest that songs and calls may be subject to partially independent selective pressures. Therefore, comparative studies examining the spatial and temporal variation of different bird vocalizations may provide valuable insights into ecological and evolutionary processes, such as adaptation, communication complexity, and population diversification, as well as into the origin and function of the different vocalization types (Freeberg 2006; Podos and Warren 2007; Freitas et al. 2025; Sebastián‐González and Pérez‐Granados 2025).

Vocal repertoire size, i.e., the number of distinct vocalization types or syllables an individual bird can produce, is one of the most widely used parameters for studying avian bioacoustics and behavioral ecology (Leighton and Birmingham 2021). The study of variation in vocal repertoire size within and among species provides valuable insights into the evolution of complexity in animal communication (Catchpole and Slater 2008). Estimates of avian vocal repertoire size at the individual level are available for hundreds of species (see Leighton and Birmingham 2021). Such traits have been correlated with male quality (Searcy et al. 1985; Nowicki et al. 1998), parental provisioning (Buchanan and Catchpole 2000), body measures (Kipper et al. 2006; Hesler et al. 2012), age (Kiefer et al. 2006), genetic diversity (Cueva et al. 2025), reproductive success (Hiebert et al. 1989; Robinson and Creanza 2019), and brain morphology (Devoogd et al. 1993), among others. Beyond the individual level, examining variation in population repertoire size, i.e., the number of distinct vocalization types or syllables within a population, can reveal how cultural transmission and habitat configuration shape vocal diversity of oscine birds (Laiolo et al. 2008; Briefer et al. 2010). Population‐level measures of repertoire size provide insights into processes such as song sharing, local dialect formation, and cultural drift, highlighting how both individual learning and collective dynamics contribute to the evolution of communication systems (Slabbekoorn and Smith 2002; Wang et al. 2022). Overall, variation in both individual and population repertoire sizes offers a powerful framework for understanding the interplay between communication, sexual selection, and cultural evolution in birds. Most of our current knowledge regarding avian vocal behavior is limited to songs and alarm calls (Catchpole and Slater 2008), with few studies incorporating non‐alarm calls or comparing song and calls within the same species (Marler 2004; Irwin et al. 2008; Gill and Bierema 2013). A comparative perspective aimed to understand how song and call repertoires vary within and among populations can reveal whether these signals change independently or co‐vary in response to shared constraints such as population size, habitat structure, or social organization.

Beyond the diversity of vocal signals produced by individuals or within a population, the extent to which they are shared among conspecifics, often defined as acoustic similarity, is a common metric used in behavioral studies. Vocal learning, i.e., the ability to learn or modify vocalizations by imitating sounds heard from adult tutors, occurs in many bird species. Therefore, the study of such regional dialects and of the patterns of sharing degree reflect how information spreads within and between populations, revealing the strength of social ties, the extent of adult dispersal movements, and connectivity among populations (Brown and Farabaugh 1997; Akçay et al. 2014; Vargas‐Castro 2015). For example, species with vocal learning tend to exhibit marked spatial variation in their vocalizations (i.e., dialects), whereas species whose vocal signals are largely innate tend to show more homogeneous acoustic patterns across space (e.g., Sebastián‐González and Pérez‐Granados 2025). The variations in such patterns can also reflect habitat changes, such as the loss of suitable habitat, as abrupt acoustic changes often indicate barriers to adult movement and increased isolation among groups and populations (Brown and Farabaugh 1997). For example, Laiolo and Tella (2005) demonstrated that the degree of song sharing between non‐neighboring Dupont's larks ( *Chersophilus duponti* ) tended to increase with the amount of habitat availability, and that landscape connectivity between patches was often a better driver of call similarity among males rather than the straight‐line distance between them (Laiolo and Tella 2006). As cultural transmission of vocal traits is not equally effective across space among spatially separated individuals, it may be possible to identify thresholds where social interactions and learning opportunities become limited. However, research identifying such thresholds remains scarce. Overall, the study of acoustic similarity patterns across distances can yield valuable insights into social structure and habitat connectivity, and therefore providing a useful framework for linking vocal behavior with ecological processes.

In this study, we selected the Dupont's lark as a model species because males produce both songs and territorial calls at high rates (see diel and seasonal patterns in Pérez‐Granados, Osiejuk, and López‐Iborra 2018), making it an excellent model for investigating differences in the singing and calling activity patterns in birds. Moreover, prior research has demonstrated that the communication system of the species is affected by habitat fragmentation and landscape configuration at both local and regional scales. For example, populations exhibit geographic variation in their acoustic signals (dialects, Laiolo and Tella 2005), and even locally restricted dialects among males occupying the same habitat patch (Pérez‐Granados et al. 2016). Habitat fragmentation has been shown to influence both songs (Laiolo and Tella 2005, 2007; Pérez‐Granados et al. 2016) and territorial calls (Laiolo and Tella 2006), with greater song and call acoustic similarity between neighboring males in continuous than in fragmented habitat patches (Laiolo and Tella 2006, see dispersal movements in Section 2.1). The different learning phases and functions proposed for territorial calls (learned before dispersal and used to advertise male identity within territories, Laiolo et al. 2007, 2008) and songs (learned after dispersal and used in a sexual context during breeding, Pérez‐Granados et al. 2016; Laiolo et al. 2008, see Section 2.1) make Dupont's lark a good candidate species to assess differences in singing and calling behavior, although there is a lack of comparative studies about whether songs and territorial calls respond similarly to habitat fragmentation and landscape configuration, or whether they convey complementary information about population processes. Likewise, there is limited knowledge regarding the extent and timing of dispersal movements in the species, particularly during the juvenile stage, which may limit our ability to interpret the patterns found. Remarkably, a study on Dupont's lark demonstrated for the first time that a cultural trait, male song repertoire size, can be potentially used as a predictor of population viability, given its positive association with annual population growth rate, productivity, and population size (Laiolo et al. 2008). However, this key finding has not been validated in subsequent years to assess its potential as an early‐warning signal of population decline.

Here, we investigated the singing and calling patterns of Dupont's lark across individuals, populations, and spatial scales, to improve our understanding of vocal behavior in steppe birds and the ecological significance of its variation. Specifically, we aimed to: (1) describe and compare song and call repertoire sizes at individual and population levels, assessing how population size and geographic area influence both vocalization types; (2) examine and compare the spatial structure of acoustic similarity in songs and calls, identifying potential thresholds of cultural transmission; and (3) evaluate whether a vocal trait, individual song repertoire size, predicts long‐term population persistence, thereby testing the potential for this trait as an indicator of population viability (Laiolo et al. 2008). Accordingly, we hypothesized that: (1) individual and population song repertoires will be larger and more variable in populations with a larger number of territories because songs are learned after dispersal, and depend on the number of available tutors. Calls, which are learned before dispersal during a short early phase, will show limited variation across individuals and populations; (2) calls will exhibit weaker spatial structuring than songs due to their pre‐dispersal learning and higher temporal stability (Laiolo and Tella 2006; Laiolo et al. 2007), resulting in more homogeneous spatial patterns and narrower cultural transmission thresholds; and (3) populations whose individuals historically exhibited smaller song repertoires will have experienced a higher probability of extinction than those with larger song repertoires (Laiolo et al. 2008). By explicitly comparing singing and calling behavior within the same study system and at different spatial scales, our study seeks to deepen our understanding of the ecological and cultural dimensions of acoustic communication in birds, to provide new insights into the singing and calling behavior of birds, and to highlight the value of bioacoustics as a tool for biodiversity conservation and monitoring threatened birds.

## 2 Methods

### 2.1 Study Species

Dupont's lark is a resident and territorial passerine inhabiting shrub‐steppes of Spain and North Africa. The Spanish (whole European) population is currently estimated around 3116 breeding males (Traba et al. 2025). The species is cataloged as Vulnerable at European scale (BirdLife International 2021) and as Endangered in Spain, since its populations are declining and largely composed of small, highly fragmented populations (Reverter et al. 2023; Traba et al. 2025). Adults exhibit limited breeding dispersal movements, commonly around 100–150 m within patches (Laiolo et al. 2007; Pérez‐Granados et al. 2022), although occasional long‐distance dispersal movements (> 75 km) indicate a certain capacity for connectivity among fragmented populations (García‐Antón et al. 2015; Navalpotro et al. 2025). Our current knowledge regarding juvenile dispersal movements is anecdotal and restricted to few cases (e.g., Pérez‐Granados et al. 2022).

**FIGURE 1:**
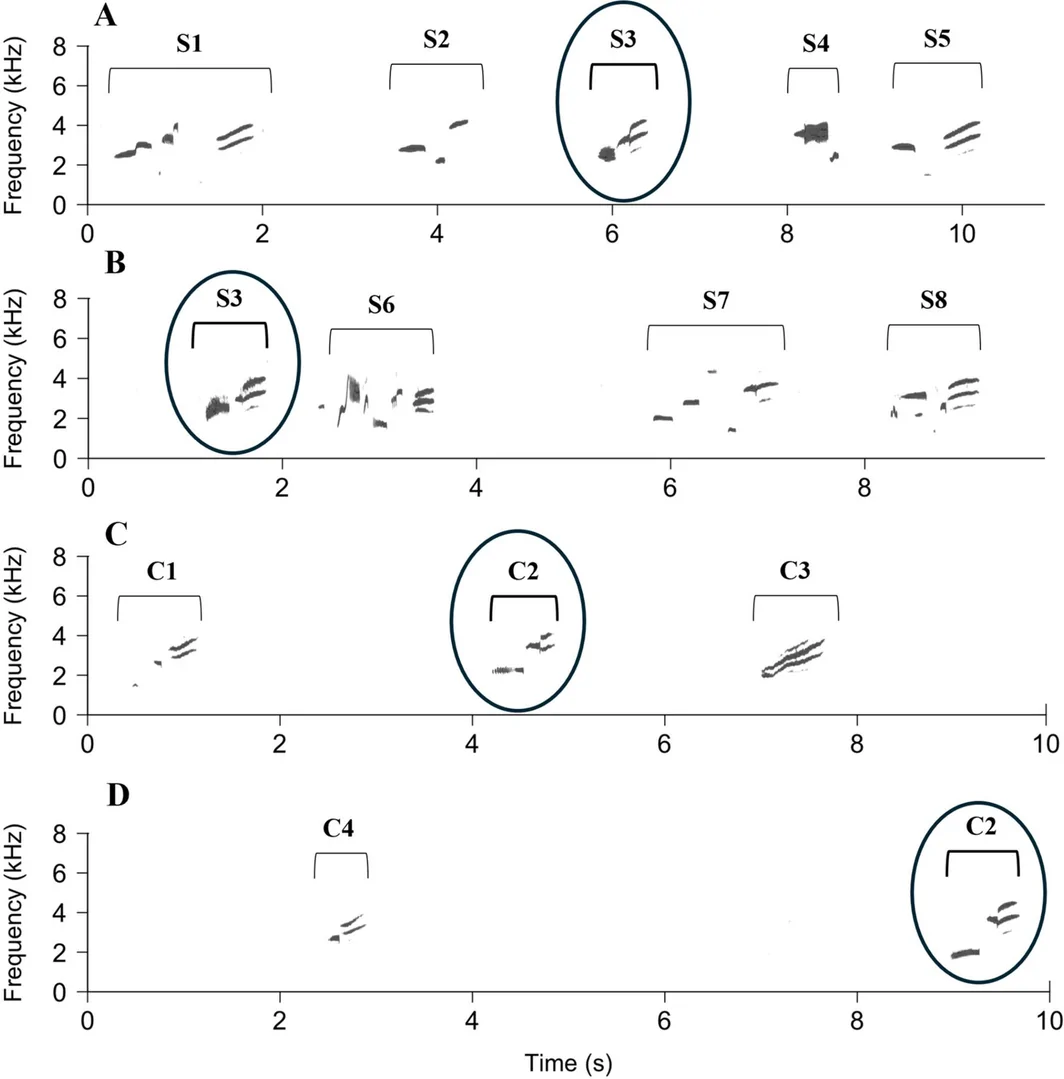
Spectrograms illustrate different song (panels A and B) and call (panels C and D) types of four Dupont's lark males recorded in different populations. Distinct song and call types are delimited by braces and identified with S for songs, C for calls, followed by a number. Vocalizations highlighted with circles (S3 and C2) indicate song and call types shared among males from different populations.

Dupont's larks show a peak of vocal activity during the hour preceding sunrise, when males produce a rich vocal repertoire composed mainly of songs and territorial calls (Pérez‐Granados, Osiejuk, and López‐Iborra 2018). Although some female larks are known to sing or emit territorial calls, such as in the Woodlark ( *Lullula arborea* ) and the Eurasian skylark ( *Alauda arvensis* , Garamszegi et al. 2007; Odom and Benedict 2018), there is no such evidence for Dupont's lark (Pérez‐Granados, Osiejuk, and López‐Iborra 2018). In the study species, songs are learned after dispersal, from nearby adult males (Laiolo and Tella 2007; Pérez‐Granados et al. 2016) and typically consist of 3–8 discrete types, each comprising 2–4 units emitted in a fixed sequence. Prior research has proposed males are age‐limited song learners, or able to only slightly modify their songs from year to year (Pérez‐Granados et al. 2016). Despite this temporal stability, the available literature has not demonstrated reliable individual identification based on song repertoires or their spectrotemporal features. Territorial calls (hereafter, calls), which have been proposed to be learned before dispersal after a short learning phase in summer (Laiolo et al. 2007), consist of 1–3 short whistles (i.e., call types) repeated regularly (Figure 1). Calls are characterized by high within‐individual repeatability and temporal stability in their spectrotemporal features, which has enabled the individual identification of Dupont's lark males (Laiolo and Tella 2006; Laiolo et al. 2008; Vögeli et al. 2008). Given the differences in the timing of call and song learning, potential tutors for call learning (before dispersal) are expected to be mainly parents and other neighboring males within the natal patch, whereas song learning, which occurs after dispersal, may involve males outside the natal patch, including neighboring or more distant males.

### 2.2 Study Area

**FIGURE 2:**
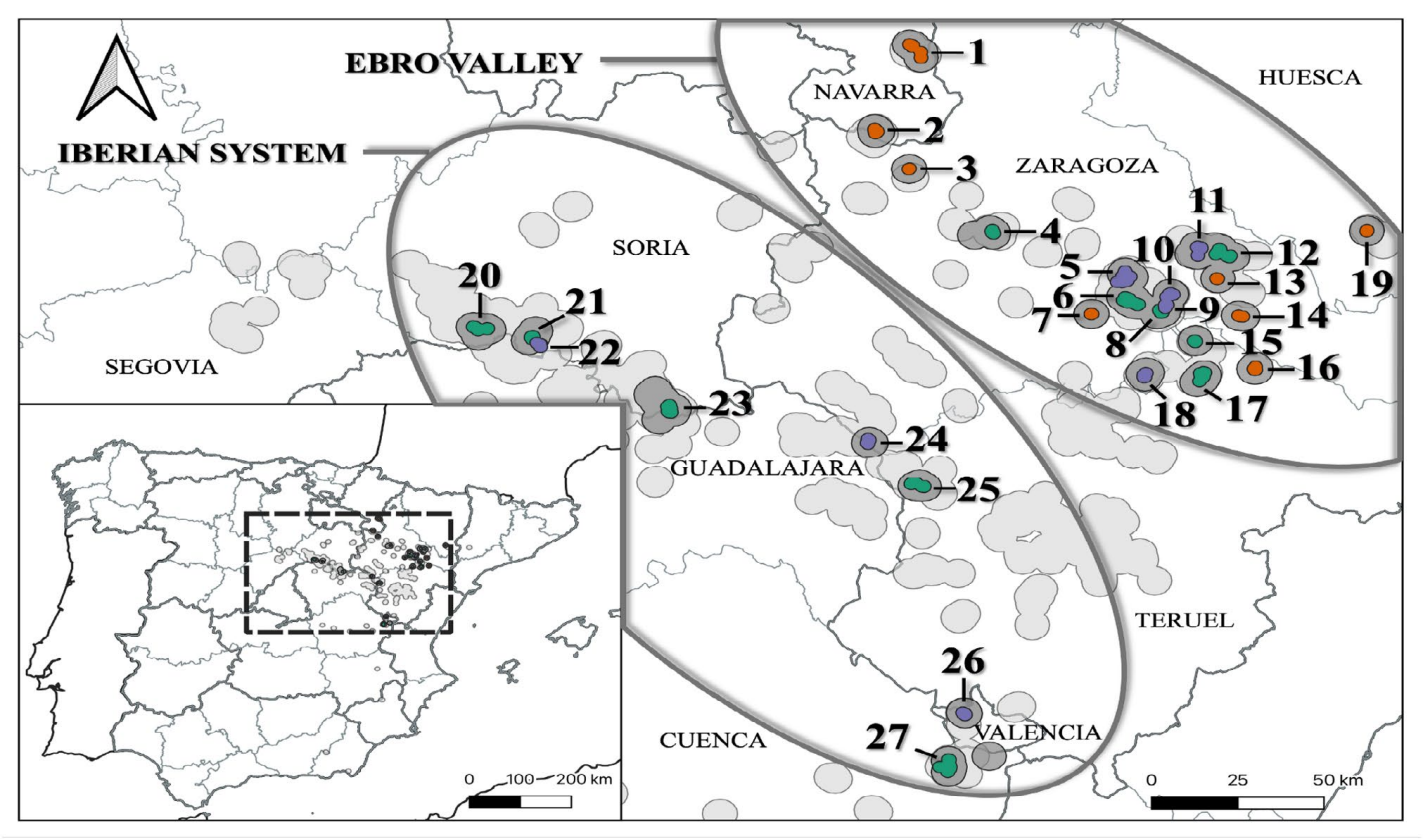
Map of studied populations. Light gray shading indicates the current distribution range of Dupont's lark in Spain (Traba et al. 2025). Green circles represent populations with more than five males recorded for both songs and calls; purple circles indicate populations with more than five males recorded for songs, but fewer than five males recorded for calls; red circles represent populations monitored but with no birds recorded. Numbers correspond to the population id as shown in Appendix 1.

The study was conducted between March and June—coinciding with the breeding season of Dupont's lark (Pérez‐Granados et al. 2017)—during 2024 and 2025. We monitored 27 habitat patches historically occupied by the species in the two main broad geographic areas in Spain (Ebro Valley and Iberian System). All patches were separated by more than 1 km of non‐suitable habitats for the species (Figure 2). Each habitat patch, hereafter referred to as a “population,” consisted of shrub‐steppe habitats interspersed with occasional trees and cereal croplands, and was visited only 1 year (Ebro Valley in 2024, Iberian System in 2025). Populations were selected to cover most of the European range of the species and to represent a gradient in population size, population density, and spatial isolation. Population area varied from 14 to 1903 ha, and local abundance of breeding Dupont's lark males in occupied sites during the study period (estimated through mapping method; Pérez‐Granados and López‐Iborra 2017) ranged from 3 to 77 individuals (see detailed data in Appendix 1). Each population was surveyed between two and five times depending on their amount of habitat available and population size. For those populations with no individuals detected during any of the visits, autonomous sound recorders (Song Meter Micro) were deployed and programmed to record during the hour before sunrise for at least 2 days to confirm absence of the species, after analyzing the recordings. This protocol has demonstrated to be effective for confirming presence or absence of Dupont's lark at population scale (e.g., Pérez‐Granados, Bustillo‐De La Rosa, et al. 2018; Pérez‐Granados, Bota, et al. 2018).

### 2.3 Bird Recording

All populations were surveyed during the hour preceding sunrise, when the species shows maximum vocal activity. Males were recorded within their territories from the closest possible distance (10–70 m) without disturbing their natural vocal behavior. Singing and calling males were recorded using a Sennheiser ME‐67 shotgun directional microphone (frequency response 50–20,000 Hz, with wind protection) and a K6 powering unit connected to either a Marantz PMD 661 or a TASCAM DR40 recorder. Recordings were made in .wav format at a sampling rate of 44.1 kHz and 24‐bit depth. Data were collected only under favorable weather conditions (i.e., no rain and calm wind). The estimated position of each recorded male was georeferenced using a GPS device (accuracy 3–5 m). Although recorded birds could not be individually identified—a task particularly challenging at night—all recordings of a given male, as well as those obtained from males within a 500 m radius, were taken on the same day to minimize the likelihood of re‐recording the same individuals on successive days. When more than one bird sang in the same area and the identity of the recorded individuals could not be confirmed, only the highest‐quality recording from a single individual was retained for analyses to avoid pseudoreplication. All recordings used in this study are available in Pérez‐Granados, Alonso‐Moya, et al. (2026).

We recorded at least 2 min of singing behavior per male, a duration considered sufficient to capture the complete individual song repertoire for this species (Pérez‐Granados, Osiejuk, and López‐Iborra 2018). In total, we recorded 188 singing males (Appendix 1). Four of these (2.2%) had slightly shorter recordings (mean of 94 s) but were retained since the typical Dupont's lark song lasts only 15–20 s. We recorded a minimum of seven different singing males (mean = 9.9, range 7–15), in 19 of the populations considered. For calling behavior, we recorded a minimum of three calls per individual, following prior research indicating that this number is adequate to capture an individual's entire call repertoire (Laiolo and Tella 2006). Recordings not meeting this criterion were excluded. Only populations with at least five recording males were retained for analyses, which was achieved in 11 populations (mean of 6.7 males, range 5–9, Figure 2). In total, we recorded 74 calling males (Appendix 1).

### 2.4 Recording Analyses

Recordings were scanned by a single expert observer (C.D.A.‐M.) using visual inspection of spectrograms in Raven Pro 1.6 (Cornell Lab of Ornithology 2023), and default configuration parameters (Window type = Hann, Discrete Fourier transform (DFT) size = 512 samples, brightness = 50, contrast = 50). The identification of song and call types within the recordings involved the precise delineation of the starting and end times, using a selection box, as well as the maximum and minimum frequency of each vocalization. All vocalizations of sufficient quality to be visually identified, and for which the observer was certain they belonged to the target individual, were included in the analyses. Background vocalizations or those produced by neighboring (non‐target) males were discarded. The annotations used in this study, available in both Raven Pro and Audacity formats, can be found in Pérez‐Granados, Alonso‐Moya, et al. (2026).

The characterization of different song and call types in the recordings was carried out blindly with respect to male ID and recording locality. Each identified song or call type was coded with an ordinal number (e.g., S1, S2 or S3 for song types, and C1, C2 or C3 for call types; Figure 1). Any song or call type that had previously been cataloged was labeled accordingly when it was found again (Figure 1). If new song or call types were identified, they were assigned a new label and added to the catalog. Following prior research on the species, the classification of song and call types was based on visual inspection of the spectrogram morphology, temporal patterns, frequency characteristics, and the number of notes (see Laiolo and Tella 2005, 2006; Pérez‐Granados, Osiejuk, and López‐Iborra 2018). When the classification of a song or call type was uncertain, or when it was annotated in two different populations, at least one additional experienced ornithologist reviewed the relevant spectrograms and audio files. The vocalization type was only confirmed when at least two researchers reached agreement.

### 2.5 Vocal Behavior Metrics

In total, we identified 396 different song types from 4208 annotated songs recorded from 188 singing males and 60 different call types from 634 annotated calls recorded from 74 calling males. Once all songs and calls were classified, two binary matrices (one for songs and one for calls) were constructed, scoring the presence (1) or absence (0) of each song or call type within the recording of each individual. We first estimated song and call repertoire sizes as the number of song and call types present in an individual's repertoire (individual repertoire size) and within a population (population repertoire size, Laiolo and Tella 2006, 2007). Secondly, we used both binary matrices to quantify the degree of song and call acoustic similarity between males, using the Jaccard similarity index (Meyer and Buchta 2019; Pérez‐Granados, Osiejuk, and López‐Iborra 2018). Finally, we estimated the geographic (Euclidean) distances between pairs of singing and calling males from their recording locations for subsequent analyses.

### 2.6 Statistical Analyses

Singing and calling behavior in the Dupont's lark were analyzed at three different scales: the individual, the population, and the geographic level (Laiolo and Tella 2006). At the individual scale, we fitted two independent, generalized linear mixed models (GLMMs, Poisson error distribution) using individual song or call repertoire size as the response variable. Geographic area (Ebro Valley/Iberian System) of the population and population size (number of territories, *z*‐standardized) were included as fixed effects to test for differences among regions and to assess the influence of population size on individual repertoires. Population identity was included as a random intercept to account for non‐independence among individuals from the same population. We did not assess the differences among populations owing to the reduced number of males recorded per population. Site area (amount of natural vegetation in the habitat patch, in hectares) was not included on the models owing to high correlation (*r* > 0.7 for both songs and calls) with population size, thereby avoiding redundancy (Dormann et al. 2013). At the population scale, we also fitted two independent generalized linear models (GLMs), using a negative binomial Type 2 distribution (“nbinom2” in R, the distribution family with best fit according to AIC values) with a log link, to analyze whether population song or call repertoire size (response variable) was related to geographic area (Ebro Valley/Iberian System), population size and number of males recorded (z‐standardized). This approach allowed us to estimate the effect of population size while controlling for sampling effort. Site area was again not included as a variable on the GLMs owing to high correlation (*r* > 0.7) with population size. Population size and number of males recorded showed very low correlation for both vocalization types (*r* < 0.18 and *p* > 0.45 for both songs and calls). GLMMs and GLMs were fitted in R (v.4.2.2, R Development Core Team 2024) using the “glmmTMB” package (Brooks et al. 2017). Model diagnostics, including checks for overdispersion, were performed using the package “DHARMa” (Hartig 2023).

We also analyzed the spatial structure of song and call acoustic similarity among males as a function of distance. Pairwise comparisons between males were divided into three discrete distance classes: (1) “Neighboring males” (pairs recorded within a 500 m radius), (2) “Non‐neighboring males” (pairs recorded farther than 500 m but within the same population), and (3) “Other localities” (pairs inhabiting different populations). The 500 m threshold was selected to facilitate direct comparisons with previous research that used the same threshold to distinguish neighboring and non‐neighboring Dupont's lark males (Pérez‐Granados et al. 2016) and because breeding dispersal movements of adult Dupont's larks are generally very limited (< 4%) beyond this distance (Garza et al. 2005; Laiolo et al. 2007; Pérez‐Granados et al. 2022). Therefore, we considered 500 m a sufficiently broad spatial category to identify potential interactions among neighboring males. For each distance class we estimated the mean similarity of all pairwise comparisons, along with the corresponding 95% confidence interval (95% CI). To test whether observed song or call acoustic similarity differed from spatially random expectations, we applied a permutation procedure based on Monte Carlo simulations (1000 permutations) using the R “boot” package (Canty and Ripley 2022) for bootstrap confidence intervals. For each distance class, a null distribution of mean acoustic similarity values was generated by randomly reshuffling male identities, and the 95% CI of the null distribution was used to assess significant deviations from random. Additionally, to better characterize the spatial decay of song and call acoustic similarity we compared the mean song and call acoustic similarity and 95% CI across discrete distance classes of 100 m, ranging from 0 to 1000 m. For this analysis, we restricted the dataset to pairs separated by 0–1000 m before conducting the permutation procedure. We identified the first distance class where the 95% CI did not overlap with that of the previous class as an approximate threshold indicating a potential change in the spatial pattern of acoustic similarity. This approach was followed to provide an estimate of the approximate range beyond which acoustic transmission or social influence between males is reduced.

To evaluate whether individual song repertoire size of Dupont's lark could predict long‐term population persistence, we fitted a GLM with binomial error distribution and logit‐link function in R (version 4.4.2). We used the historical individual song repertoire sizes estimated in 50 populations occupied by the species and monitored between 2004 and 2007 (Laiolo et al. 2007, 2008; Vögeli et al. 2010). We considered the 18 Dupont's populations from the Ebro Valley analyzed by Laiolo et al. (2008), the study where the hypothesis was proposed, but we also considered data collected using the same methodology from 32 additional populations spanning most of the European distribution of the species (Laiolo et al. 2008; Vögeli et al. 2010). Individual song repertoire sizes were estimated following the same methodology in all populations (see Laiolo et al. 2008). In the GLM, population fate in 2025 (presence = 1, absence = 0) was used as the response variable, and the original values of mean individual song repertoire size, extracted from the original studies, for each population monitored as the main predictor (Laiolo et al. 2007, 2008). Population fate was obtained using the most updated information regarding the species distribution in Spain (Traba et al. 2025, authors' own data). A population was considered absent (i.e., extinct) when at least two surveys between 2004 and 2025, including 2025, detected no individuals.

## 3 Results

**FIGURE 3:**
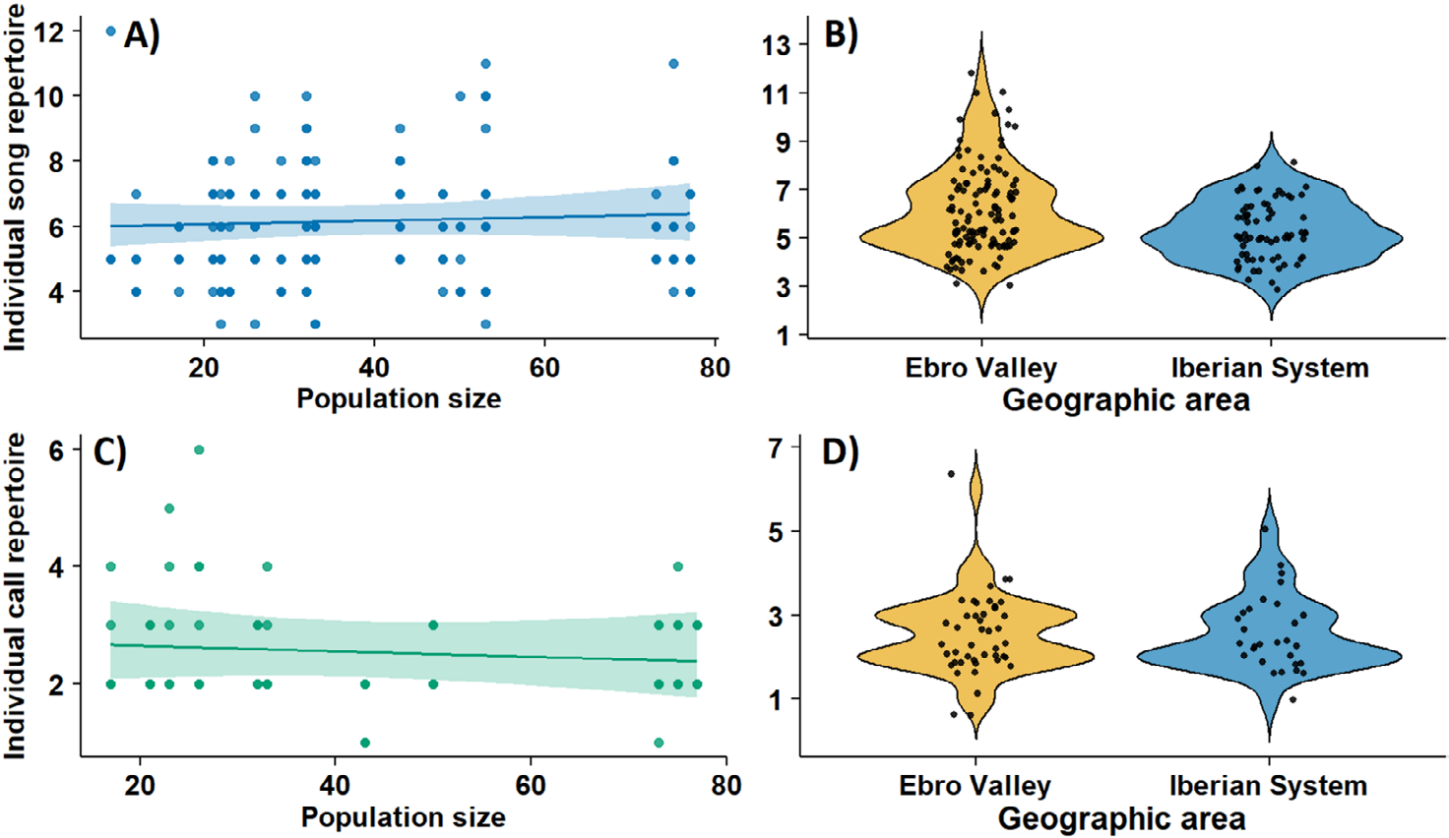
Individual acoustic repertoire size of Dupont's lark across population sizes and geographic areas. Panels on the left show the relationship between individual song (A, top) and call (C, bottom) repertoire size and population size. Points represent the observed values, solid lines show model‐predicted means, and shaded bands indicate 95% confidence intervals. The panels on the right show violin plots of individual song (B) and call (D) repertoire size across geographic areas.

**FIGURE 4:**
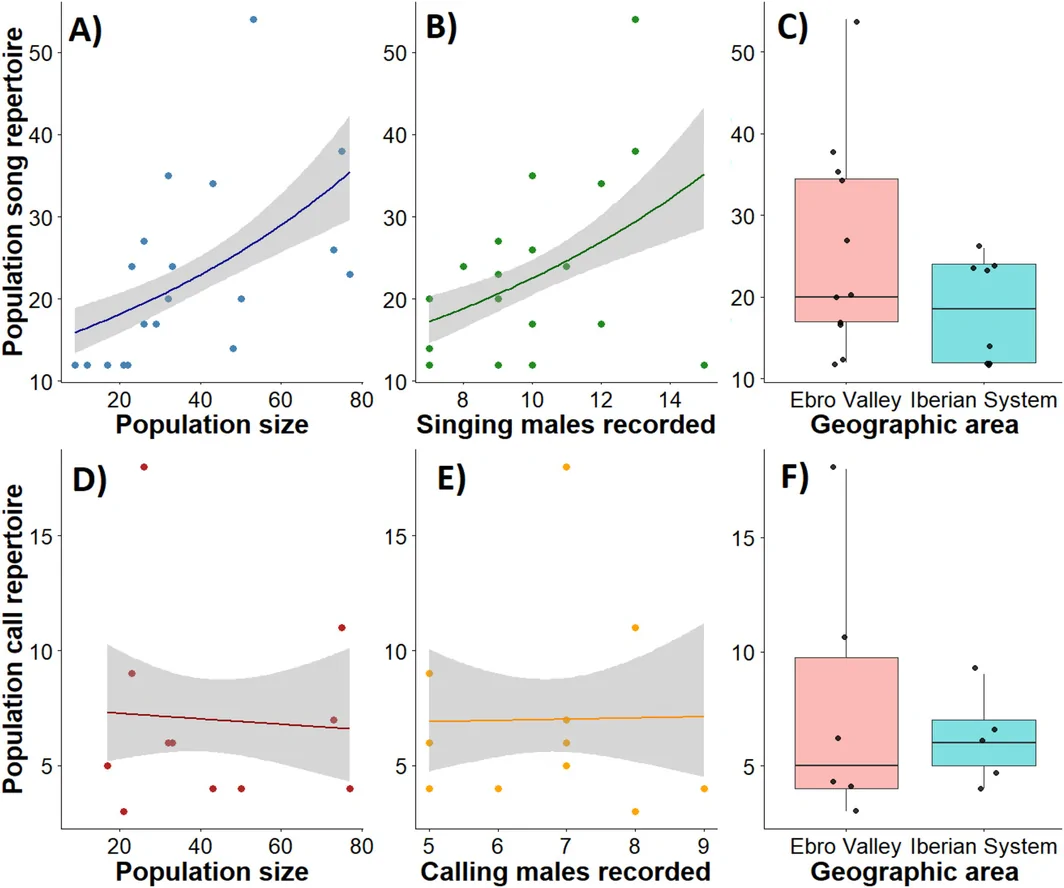
Relationships between population repertoire size and population size and number of males recording for song (A, B) and call (D, E). Points represent the observed values, solid lines represent model predictions, and shaded areas indicate 95% confidence intervals. Boxplots (C–F) summarize distributions of population song and call repertoire size across geographic areas.

According to the GLMMs, individual song repertoire size was not significantly related to population size (estimate = 0.018, SE = 0.030, *z* = 0.59, *p* = 0.556; Figure 3A), but significantly varied among geographic areas (estimate = −0.150, SE = 0.064, *z* = −2.36, *p* = 0.018). This indicates that birds from the Ebro Valley had larger repertoires than those from the Iberian System (Figure 3B). Neither population size (estimate = −0.040, SE = 0.075, *z* = −0.54, *p* = 0.588) nor geographic area (estimate = 0.006, SE = 0.150, *z* = 0.04, *p* = 0.967) had a significant relationship with individual call repertoire size (Figure 3C,D). The GLMs showed that population song repertoire size was significantly related to population size (estimate = 0.245, SE = 0.079, *z* = 3.07, *p* = 0.002), indicating that larger populations tend to have larger song repertoires (Figure 4A). However, neither the number of singing males recorded (estimate = 0.102, SE = 0.089, *z* = 1.15, *p* = 0.248) nor the geographic area of the population (estimate = −0.281, SE = 0.168, *z* = −1.68, *p* = 0.093) were significantly related to population song repertoire (Figure 4B,C). For population call repertoire size, neither population size (estimate = −0.012, SE = 0.158, *z* = −0.08, *p* = 0.939), the number of call males recorded (estimate = −0.114, SE = 0.244, *z* = −0.46, *p* = 0.642), nor the geographic area of the population (estimate = −0.351, SE = 0.446, *z* = 0.79, *p* = 0.431) had a significant relationship with call repertoire sizes (Figure 4D–F).

**FIGURE 5:**
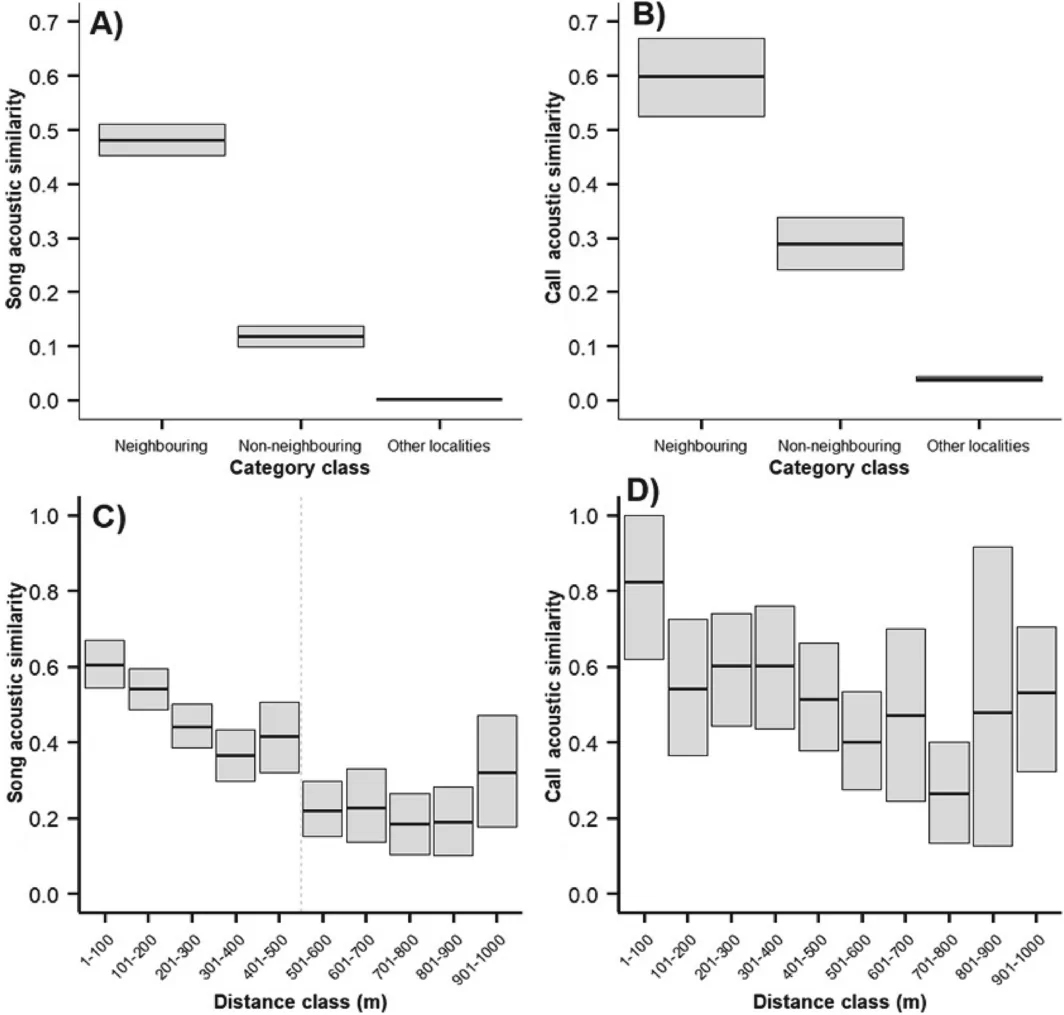
Acoustic similarity among pairs of Dupont's lark males across space. Panels show the mean (horizontal black line) and 95% confidence intervals (gray boxes) for song (left column) and call (right column) acoustic similarity across space. Subpanels show: Song (A) and call (B) acoustic similarity of Dupont's lark males across three distance categories; song (C) and call (D) acoustic similarity across 100‐m distance range categories. For all panels the full statistics, including the expected similarity values under the observed and random distribution, and the number of male pairs contributing to each comparison, are provided in Appendix 2.

Song and call acoustic similarity among males decreased sharply with spatial distance, although call acoustic similarity was always higher than song acoustic similarity for each category or 100 m distance range analyzed (Figure 5). For both vocalization types, neighboring males (< 500 m) showed the highest acoustic similarity (SONGS: mean = 0.48; 95% CI: 0.45–0.51; CALLS: mean = 0.599; 95% CI: 0.526–0.674), and non‐neighboring males within the same population exhibited intermediate similarity values (SONGS: mean = 0.120; 95% CI: 0.10–0.14; CALLS: mean = 0.288; 95% CI: 0.243–0.336), in both cases showing significantly higher values than expected by chance (Figure 5A,B, see full statistics in Appendix 2). Males from different localities displayed almost no song (mean = 0.0015; 95% CI: 0.0013–0.0016) or call acoustic similarity (mean = 0.039; 95% CI: 0.034–0.044), significantly lower than expected by chance (Appendix 2). Fine‐scale analyses using 100 m distance range revealed distinct spatial structuring for the two vocalization types. Song acoustic similarity declined steadily across 100 m distance range categories, stabilizing at low values beyond ~500 m, where 95% CIs of consecutive distance range categories no longer overlap with values < 500 m (Figure 5C). In contrast, call acoustic similarity remained relatively constant across all 100 m distance ranges, with overlapping 95% CIs up to 1000 m (Figure 5D).

**FIGURE 6:**
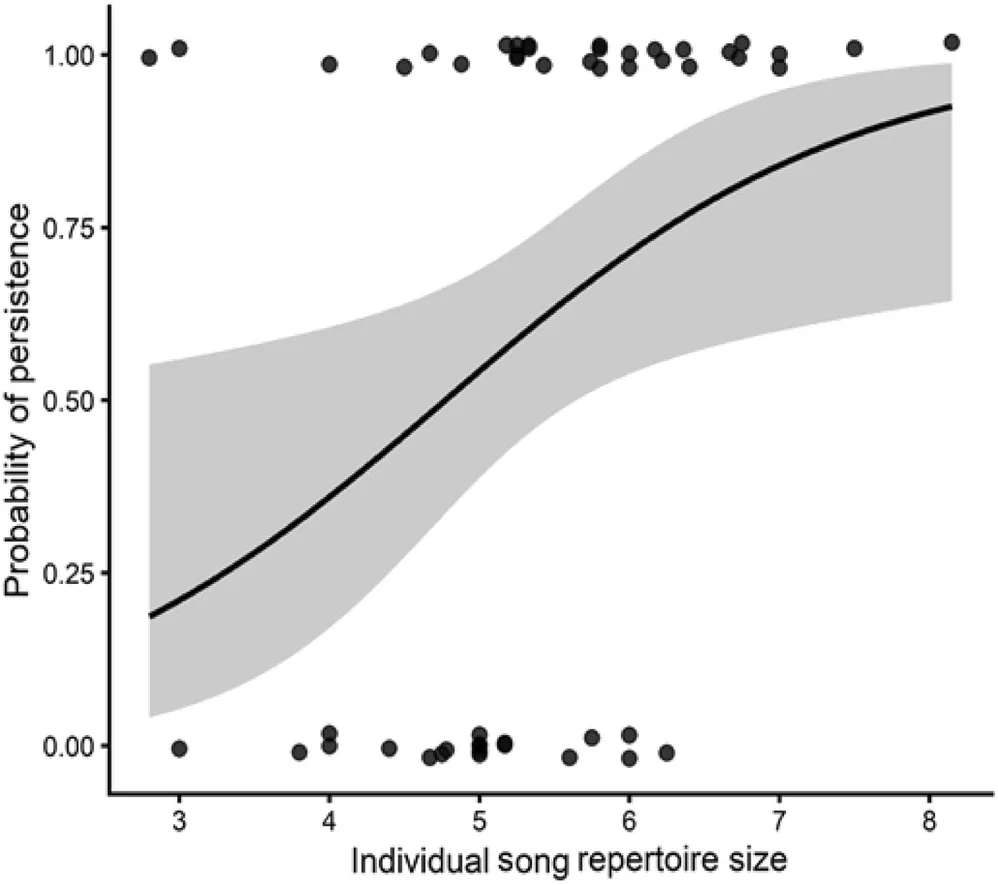
Logistic regression curve showing the probability of long‐term population persistence of Dupont's lark as a function of mean individual song repertoire size recorded during 2004–2007. The solid black line represents the fitted model within the range of observed predictor values, while the shaded gray area indicates the 95% confidence interval. Points represent the observed fate (present or extinct) of the 50 populations monitored in 2025, with minimal fitter applied to improve visual separation.

The binomial logistic regression revealed that mean individual song repertoire size was significantly related to long‐term population persistence (*β* = 0.745 ± 0.324 SE, *z* = 2.30, *p* = 0.021). The model shows that Dupont's lark populations with larger individual song repertoires had a significantly higher probability of persisting until 2025 (Figure 6).

## 4 Discussion

Here, we provide one of the first assessments comparing the song and call behavior of a bird species, and whether both vocalization types were influenced by individual, population, and landscape factors. Overall, we found a clear divergence between the ecological and social factors influencing both vocalization types, in agreement with prior research that also reported differences in the daily and seasonal patterns of both vocalization types (see Pérez‐Granados, Osiejuk, and López‐Iborra 2018), and that suggested that songs and calls may serve distinct functions on Dupont's lark communication. At the individual level, song repertoire size varied among geographic areas, with males from the Ebro Valley exhibiting slightly larger repertoires than those from the Iberian System. Although we cannot completely rule out potential year effects (Ebro Valley populations were recorded in 2024 and Iberian System in 2025), previous research on this species found similar song type diversity between years within the same locations (Pérez‐Granados et al. 2016), suggesting that temporal variation is unlikely to be a major factor explaining the geographical pattern found here. However, contrary to previous research (Laiolo and Tella 2007; Laiolo et al. 2008), we found no significant relationship between individual song repertoire size and population size. Such a relationship had been proposed because males surrounded by a larger number of neighbors were more likely to increase their repertoire through imitative singing (Laiolo and Tella 2007). It is worth highlighting that all populations monitored in the current study had more than nine males, with a mean population size of 38 (Appendix 1). Laiolo et al. (2008) suggested that the relationship between the acoustic behavior of Dupont's lark and population size might be non‐linear; for example, no variation in vocal activity rate was observed in populations with more than nine males. Therefore, the larger population sizes in our study may likely explain differences with previous research, which included nine populations (most of which are already extinct) with a maximum of nine males (Laiolo et al. 2008). In contrast to song repertoire size, we found no relationship between individual call repertoire size and geographic area, suggesting that calls might be a more conservative vocalization type than songs, with little variation among populations.

At the population level, song repertoire size increased with population size, in agreement with previous research (Laiolo and Tella 2007). Importantly, the number of singing males recorded per population was not correlated with the overall estimated population size (Pearson correlation, *r* = 0.18, *p* = 0.45), and the number of singing males recorded per population remained relatively constant among populations (9.9 ± 0.5, Mean ± SE, range 7–15). We suggest that the larger song repertoires observed in patches with larger population sizes are likely explained by the presence of multiple dialects among groups of males inhabiting the same habitat patch (i.e., neighboring males shared several song types among them, but not with non‐neighboring groups, see also Pérez‐Granados et al. 2016). Again, population call repertoires did not vary with population size, geographic area, or with the number of calling males recorded. Overall, our results regarding repertoire sizes support the notion that songs are more plastic and culturally transmitted traits in Dupont's lark males, which can diverge between regions and populations through local learning and social interactions, whereas calls appear to be more stable.

Prior research has proposed that Dupont's lark males learn to sing from nearby adult males after their dispersal movements (Pérez‐Granados et al. 2016), whereas calls are learned before dispersal (Laiolo and Tella 2005; Laiolo 2008). Therefore, comparing the spatial structure of songs and calls shared among males and between populations can provide insights into the dispersal behavior of both adult and juvenile birds. We found high acoustic similarity in both songs and calls among males within the same habitat patch, particularly between neighboring males (< 500 m apart), but none among different patches. Our results also show that song transmission among males is largely restricted to distances about 500 m, a threshold that may indicate the most common dispersal movements of adult birds, which agrees with prior research regarding adult breeding dispersal movements by the species (Laiolo et al. 2007; Pérez‐Granados et al. 2022). In contrast, call acoustic similarity was more homogeneous within 1 km, suggesting that juvenile Dupont's larks may perform movements of at least 1 km within their natal habitat patch, before dispersal. Our current knowledge of the species' dispersal ability is very limited and almost restricted to adult males (Laiolo et al. 2007; Vögeli et al. 2008; Pérez‐Granados et al. 2023), with a complete lack of information about the movement ecology of juveniles prior to dispersal. The low breeding movement distances described for adult males (ca. 150–200 m; Laiolo et al. 2007; Pérez‐Granados et al. 2022) agree with the high acoustic song similarity between males within patches (Beecher et al. 1997; Hill et al. 1999). The lower spatial structure of call acoustic similarity within patches, compared to songs, suggests that juvenile birds move within their natal patches before dispersal. Overall, the 100‐m distance‐classes patterns described here should be interpreted as descriptive and with caution, particularly given the relatively low number of pairwise comparisons for call similarity (< 20 pairs per distance class). Nonetheless, the patterns identified provide new knowledge regarding the ecology of the target species and about how song and call acoustic similarity patterns may differ over space. Further studies with larger sample sizes, particularly for calls, will be needed to confirm the patterns found.

Our results provide strong support for the role of song repertoire size as an indicator of population persistence, in line with the hypothesis proposed by Laiolo et al. (2008). We found a significant association between the mean individual song repertoire within a population and its probability of persistence, with extinct populations exhibiting smaller repertoire sizes (mean of 4.9, *N* = 20) compared to those that persisted over time (mean of 5.7, *N* = 30). In fact, only one extinct population (5%) showed a mean repertoire size above 6.0, whereas 13 extant populations (43%) exceeded this value. Notably, none of the nine populations with mean repertoire sizes above 6.25 became extinct over the following two decades, suggesting this value as a potential threshold below which the probability of a population to become extinct increases markedly. Our dataset expands substantially the results presented by Laiolo et al. (2008). We included the 18 Dupont's lark populations from the Ebro Valley analyzed by Laiolo et al. (2008), but we also considered data from 32 additional populations spanning most of the European distribution of the species. This broader spatial coverage together with the analyses of population persistence two decades ago provides a robust assessment of the potential use of acoustic traits, such as individual song repertoire, as indicators of population status. We acknowledge that population persistence of Dupont's lark might be influenced by multiple interacting factors not addressed in our study, including wind farm development (Gómez‐Catasús et al. 2018), land‐use practices such extensive grazing (Gómez‐Catasús et al. 2023; Reverter et al. 2025), landscape connectivity and genetic erosion (Méndez et al. 2014; Bustillo‐de‐la‐Rosa et al. 2024), habitat quality (Vögeli et al. 2010; Gómez‐Catasús et al. 2019), initial population size (Laiolo et al. 2008), weather conditions (Pérez‐Granados, Payo‐Payo, et al. 2026), and stochastic extreme weather events (Pérez‐Granados et al. 2023). Nevertheless, our findings highlight the potential role of reduced song repertoire size as an early‐warning signal of population decline.

In this study, we assessed the ecological and demographic factors shaping the singing and calling behavior of a threatened steppe bird, improving our current knowledge on acoustic communication in this vulnerable bird group (Gómez‐Catasús et al. 2025). Overall, the song of the Dupont's lark seems to be a flexible cultural trait that varies among areas and is sensitive to population size, geographic area, spatial proximity, and connectivity, whereas calls remain more conserved and spatially stable in the species. Nonetheless, the factors shaping acoustic patterns in song and calls are likely to differ among bird species depending on their movement ecology, vocal learning abilities, and the type of vocalization considered (learned or genetically inherited). Further research integrating a wider range of vocalization types and species will be essential to clarify how cultural processes interact with ecological constraints to shape acoustic communication in birds. In this context, incorporating data on juvenile dispersal and population genetics of the Dupont's lark across its distribution range could provide deeper insight into the interplay between ecological and cultural drivers. Importantly, our findings highlight the utility of acoustic traits as practical tools for conservation. The mean individual repertoire size within a population was a significant predictor of Dupont's lark population persistence, suggesting that it may serve as a non‐invasive, early warning signal for tracking population health and informing conservation strategies for this threatened steppe bird.

## Author Contributions

**Cristian Pérez‐Granados:** conceptualization (lead), data curation (lead), formal analysis (lead), funding acquisition (lead), investigation (lead), methodology (lead), project administration (lead), resources (lead), software (lead), supervision (lead), validation (lead), visualization (lead), writing – original draft (lead), writing – review and editing (lead). **Cristina D. Alonso‐Moya:** data curation (lead), formal analysis (equal), methodology (equal), visualization (equal), writing – review and editing (equal). **Adrián Barrero:** data curation (equal), formal analysis (equal), methodology (equal), writing – review and editing (equal). **Pedro Sáez‐Gómez:** data curation (equal), formal analysis (equal), methodology (equal), writing – review and editing (equal). **Gerard Bota:** data curation (equal), formal analysis (equal), methodology (equal), writing – review and editing (equal). **José J. Lahoz‐Monfort:** data curation (equal), formal analysis (equal), methodology (equal), writing – review and editing (equal). **Paola Laiolo:** data curation (equal), formal analysis (equal), methodology (equal), writing – review and editing (equal). **María Méndez:** data curation (equal), formal analysis (equal), methodology (equal), writing – review and editing (equal). **David Serrano:** data curation (equal), formal analysis (equal), methodology (equal), writing – review and editing (equal). **José L. Tella:** data curation (equal), formal analysis (equal), methodology (equal), writing – review and editing (equal). **Matthias Vögeli:** data curation (equal), formal analysis (equal), methodology (equal), writing – review and editing (equal). **Juan Traba:** conceptualization (equal), data curation (equal), formal analysis (equal), funding acquisition (equal), investigation (equal), methodology (equal), project administration (equal), resources (equal), software (equal), supervision (equal), validation (equal), visualization (equal), writing – review and editing (equal).

## Funding

This work was supported by the Spanish Ministry of Science, Innovation and Universities (RYC2024‐048830‐I), European Commission, LIFE Connect Ricotí (LIFE20 NAT/ES/000133), Ministerio de Ciencia e Innovación of Spain (PID2022‐139294NB‐I00).

## Conflicts of Interest

The authors declare no conflicts of interest.

## Data Availability

The acoustic dataset and song and call annotations used in the study are available in https://doi.org/10.1038/s41597‐026‐07131‐4. The datasets and codes created are archived on Figshare repository and are available in https://doi.org/10.6084/m9.figshare.32390706.

## Appendix 1 Summary table indicating the ID (Pop. ID) and name of each Dupont's lark population monitored, along with patch size (ha) and population size (Pop. Size). For those populations with more than five singing or calling males recorded, and therefore included in the analyses, we provide, for each vocalization type, the number of males recorded, the overall population repertoire, and the mean individual repertoire size

**Table:**

Pop. ID	Population	Patch size	Pop. size	SONG			CALLS		
				Males recorded	Population song repertoire	Mean individual song repertoire size	Males recorded	Population call repertoire	Mean individual call repertoire size
1	Bardenas	814.1	33	—	—	—	—	—	—
2	Ablitas	117	28	—	—	—	—	—	—
3	Pozuelo	37.4	0	—	—	—	—	—	—
4	Urrea Jalón	158.7	32	10	35	7.4	7	6	2.71
5	Mediana	510.1	53	13	54	6.69		7	2.33
6	Lomaza	771.6	75	13	38	6.08	8	11	2.75
7	Puebla	38.2	0	—	—	—	—	—	—
8	El Planerón	240.0	50	7	20	5.57	9	4	2.44
9	Planerón Norte	90.2	26	12	17	5.67	—	—	—
10	Fuentes	260.8	9	7	12	6	—	—	—
11	Pina Rabasa	105.0	29	10	17	5.2	—	—	—
12	Pina Castane	486.4	43	12	34	6.5	6	4	1.5
13	Pina Lerín	27.0	3	—	—	—	—	—	—
14	Cinco Olivas	19.4	0	—	—	—	—	—	—
15	Azaila	42.1	21	15	12	6.07	8	3	2.38
16	Samper	14.6	0	—	—	—	—	—	—
17	Albalate	370.2	26	9	27	6.89	7	18	3.29
18	Lécera	126.7	32	9	20	5.44	—	—	—
19	Ballobar	23.4	0	—	—	—	—	—	—
20	Retortillo	356.1	73	10	26	5.5	7	7	2.14
21	La Riba	132.2	17	7	12	5.14	7	5	2.43
22	Barcones	393.0	48	7	14	5.71	—	—	—
23	Layna	888.0	77	9	23	5.22	5	4	2.6
24	Embid	110.5	22	9	12	5	—	—	—
25	Blancas	536.4	23	8	24	5.88	5	9	3.2
26	Hontanar	198.9	12	10	12	4.5	—	—	—
27	Moya	274.4	33	11	24	5.64	5	6	2.6

## Appendix 2 Observed and random (null) acoustic similarity among pairs of Dupont's lark males. The table summarizes the mean acoustic similarity (Observed) and range (95% confidence intervals, between brackets) for each distance category and 100‐m distance range, along with the number of observed males (Pairs). It also shows the mean acoustic similarity and range (95% confidence intervals) estimated from 1000 permutations (random). Two separate sub‐tables are presented: The top table for song and the bottom table for call acoustic similarity

**Table:**

Song acoustic similarity			
	Observed	Pairs	Random
Category			
Neighboring	0.48 (0.45–0.51)	396	0.015 (0.015–0.016)
Non‐neighboring	0.120 (0.10–0.14)	488	0.016 (0.008–0.0244)
Other localities	0.002 (0.001–0.002)	16,694	0.016 (0.015–0.016)
Distance range			
0–100	0.604 (0.543–0.669)	68	0.409 (0.339–0.479)
101–200	0.540 (0.486–0.594)	113	0.410 (0.361–0.458)
201–300	0.441 (0.384–0.502)	92	0.408 (0.346–0.466)
301–400	0.365 (0.297–0.434)	74	0.409 (0.346–0.475)
401–500	0.415 (0.319–0.505)	49	0.407 (0.328–0.486)
501–600	0.220 (0.152–0.298)	55	0.406 (0.333–0.482)
601–700	0.226 (0.137–0.330)	33	0.408 (0.308–0.510)
701–800	0.184 (0.103–0.265)	19	0.413 (0.281–0.547)
801–900	0.190 (0.100–0.282)	28	0.410 (0.298–0.524)
901–1000	0.321 (0.175–0.472)	16	0.408 (0.252–0.557)

Call acoustic similarity			
	Observed	Pairs	Random
Category			
Neighboring	0.599 (0.526–0.674)	83	0.068 (0.033–0.107)
Non‐neighboring	0.288 (0.243–0.336)	131	0.068 (0.040–0.099)
Other localities	0.039 (0.034–0.044)	2487	0.0680 (0.066–0.070)
Distance range			
0–100	0.824 (0.618–1.000)	11	0.542 (0.341–0.727)
101–200	0.541 (0.365–0.725)	17	0.542 (0.384–0.696)
201–300	0.601 (0.443–0.741)	19	0.543 (0.403–0.688)
301–400	0.603 (0.436–0.760)	17	0.539 (0.381–0.691)
401–500	0.513 (0.377–0.662)	19	0.542 (0.397–0.689)
501–600	0.400 (0.275–0.533)	10	0.543 (0.335–0.759)
601–700	0.470 (0.245–0.700)	10	0.544 (0.342–0.742)
701–800	0.265 (0.133–0.400)	8	0.543 (0.306–0.750)
801–900	0.479 (0.125–0.917)	4	0.538 (0.217–0.875)
901–1000	0.531 (0.322–0.704)	9	0.542 (0.333–0.741)
